# Supplementary material for: Diagnostic Performances of the ACR-TIRADS System in Thyroid Nodules Triage: A Prospective Single Center Study
Source: Cancers (Basel). 2021 May 6;13(9):2230. doi: 10.3390/cancers13092230 (PMC8124822; doi:10.3390/cancers13092230)
Supplement: Supplementary file 1 [file cancers-13-02230-s001.zip › cancers-1165743-supplementary.pdf]

cancers

MDPI

Supplementary Material: Diagnostic Performances of the ACR-TIRADS System in Thyroid Nodules Triage: A Prospective Single Center Study

Davide Leni, Davide Seminati, Davide Fior, Francesco Vacirca, Giulia Capitoli, Laura Cazzaniga, Camillo Di Bella, Vincenzo L'Imperio, Stefania Galimberti and Fabio Pagni

Table S1. Correlation of the histological cancer subtype, local tumor staging (pTNM, following the AJCC 8th edition), US-ACR and cytological classes (according to SIAPEC/Bethesda classifications) of the subset of cases submitted to surgery with a final diagnosis of malignancy (34/49, 70%).

N.	Histology	pTNM	ACR Classes	Cytology (SIAPEC/Bethesda)
1	EFVPTC	pT3a	3	TIR3B/IV
2	EFVPTC	pT1b	5	TIR3B/IV
3	EFVPTC	pT1b	3	TIR3A/III
4	PTC	pT2	5	TIR3A/III
5	PTC	pT1b	4	TIR3B/IV
6	PTC	pT1a	5	TIR3B/IV
7	PTC	pT1a	4	TIR4/V
8	PTC	pT1b	3	TIR4/V
9	PTC	pT1b	4	TIR4/V
10	PTC	pT1b	4	TIR4/V
11	PTC	pT3a	5	TIR4/V
12	PTC	pT1a	3	TIR4/V
13	PTC	pT1b	4	TIR4/V
14	PTC	pT2	4	TIR4/V
15	PTC	pT1b	5	TIR4/V
16	PTC	pT1b	5	TIR5/VI
17	PTC	pT1b	5	TIR5/VI
18	PTC	pT1b	5	TIR5/VI
19	PTC	pT2	5	TIR5/VI
20	PTC	pT1b	5	TIR5/VI
21	PTC	pT1b	4	TIR5/VI
22	PTC	pT1b	4	TIR5/VI
23	PTC	pT1b	5	TIR5/VI
24	PTC	pT1a	4	TIR5/VI
25	PTC	pT1b	5	TIR5/VI
26	PTC	pT1b	5	TIR5/VI
27	PTC	pT1a	5	TIR5/VI

28	PTC	pT1b	5	TIR5/VI
29	PTC	pT1b	5	TIR5/VI
30	PTC	pT1b	3	TIR5/VI
31	Minimally invasive Hurthle cell carcinoma	pT1b	5	TIR3B/IV
32	Medullary carcinoma	pT1a	4	TIR4/V
33	Anaplastic carcinoma	pT3b	5	TIR5/VI
34	Spindle cell melanoma metastasis	/	4	TIR5/VI

PTC: papillary thyroid carcinoma. EFVPTC: encapsulated follicular variant of PTC.

Table S2. Description of the evaluation made by two radiologists of the same 17 nodules.

	**N° of Discordant Cases**	**First Assessment**	**Second Assessment**
ACR class	7/17		
detailed disagreement	1/17	2	3
-	1/17	3	1
-	2/17	3	4
-	1/17	4	3
-	1/17	5	1
-	1/17	5	4
Ultrasound features			
Composition	3/17		
detailed disagreement	1/17	1	0
-	1/17	1	2
-	1/17	2	0
Echogenicity	8/17		
detailed disagreement	2/17	1	2
-	1/17	2	0
-	3/17	2	1
-	1/17	2	3
-	1/17	3	2
Shape	1/17	3	0
Echogenic foci	2/17		
detailed disagreement	1/17	1	0
-	1/17	3	1

Table S3. Comparison of the classes and relative operative indications for the three most commonly used US-based TIRADS systems (ACR, EU and ATA) for the screening of thyroid nodules.

ACR Classes	Final Indication ACR	EU Classes	Final Indication EU	ATA Classes	Final Indication ATA
ACR1	No FNA	EU1	No FNA	ATA1	No FNA
ACR2	No FNA	EU2	No FNA	ATA2	FNA or follow-up if ≥ 2 cm
ACR3	FNA if ≥ 2.5 cm<br>Follow-up if ≥ 1.5 cm	EU3	FNA if > 2 cm	ATA3	FNA if ≥ 1.5 cm
ACR4	FNA if ≥ 1.5 cm<br>Follow-up if ≥ 1 cm	EU4	FNA if > 1.5 cm	ATA4	FNA if ≥ 1 cm
ACR5	FNA if > 1 cm<br>Follow-up if ≥ 0.5 cm	EU5	FNA if > 1 cm<br>Follow-up if ≤ 1 cm	ATA5	FNA if ≥ 1 cm

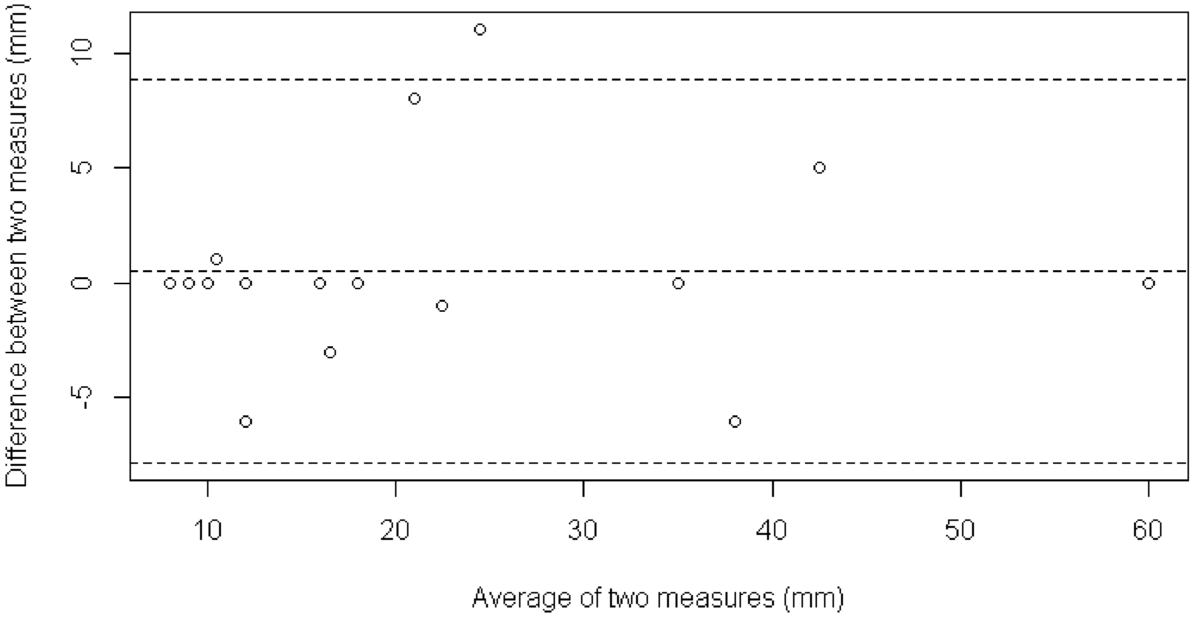

Figure S1. The graph reports the differences in measures as assessed by the Bland-Altmann analysis.
